# Selenomethionine alleviates chronic heat stress-induced breast muscle injury and poor meat quality in broilers via relieving mitochondrial dysfunction and endoplasmic reticulum stress

**DOI:** 10.1016/j.aninu.2023.12.008

**Published:** 2024-02-01

**Authors:** Jinzhong Jing, Jiayi Wang, Xiaoyu Xiang, Shenggang Yin, Jiayong Tang, Longqiong Wang, Gang Jia, Guangmang Liu, Xiaoling Chen, Gang Tian, Jingyi Cai, Bo Kang, Lianqiang Che, Hua Zhao

**Affiliations:** a Key Laboratory for Animal Disease-Resistance Nutrition of Ministry of Education, of China Ministry of Agriculture and Rural Affairs, of Sichuan Province, Animal Nutrition Institute, Sichuan Agricultural University, Chengdu 611130, Sichuan, China; b College of Animal Science and Technology, Sichuan Agricultural University, Chengdu 611130, Sichuan, China

**Keywords:** Broiler, Chronic heat stress, Skeletal muscle, Meat quality, Mitochondrial dysfunction, Endoplasmic reticulum stress

## Abstract

In the present study, the chronic heat stress (CHS) broiler model was developed to investigate the potential protection mechanism of organic selenium (selenomethionine, SeMet) on CHS-induced skeletal muscle growth retardation and poor meat quality. Four hundred Arbor Acres male broilers (680 ± 70 g, 21 d old) were grouped into 5 treatments with 8 replicates of 10 broilers per replicate. Broilers in the control group were raised in a thermoneutral environment (22 ± 2 °C) and fed with a basal diet. The other four treatments were exposed to hyperthermic conditions (33 ± 2 °C, 24 h in each day) and fed on the basal diet supplied with SeMet at 0.0, 0.2, 0.4, and 0.6 mg Se/kg, respectively, for 21 d. Results showed that CHS reduced (*P <* 0.05) the growth performance, decreased (*P <* 0.05) the breast muscle weight and impaired the meat quality of breast muscle in broilers. CHS induced protein metabolic disorder in breast muscle, which increased (*P <* 0.05) the expression of caspase 3, caspase 8, caspase 9 and ubiquitin proteasome system related genes, while decreased the protein expression of P-4EBP1. CHS also decreased the antioxidant capacity and induced mitochondrial stress and endoplasmic reticulum (ER) stress in breast muscle, which increased (*P <* 0.05) the ROS levels, decreased the concentration of ATP, increased the protein expression of HSP60 and CLPX, and increased (*P <* 0.05) the expression of ER stress biomarkers. Dietary SeMet supplementation linearly increased (*P <* 0.05) breast muscle Se concentration and exhibited protective effects via up-regulating the expression of the selenotranscriptome and several key selenoproteins, which increased (*P* < 0.05) body weight, improved meat quality, enhanced antioxidant capacity and mitigated mitochondrial stress and ER stress. What's more, SeMet suppressed protein degradation and improved protein biosynthesis though inhibiting the caspase and ubiquitin proteasome system and promoting the mTOR-4EBP1 pathway. In conclusion, dietary SeMet supplementation increases the expression of several key selenoproteins, alleviates mitochondrial dysfunction and ER stress, improves protein biosynthesis, suppresses protein degradation, thus increases the body weight and improves meat quality of broilers exposed to CHS.

## 1 Introduction

At present, broilers possess the characteristics of rapid growth rate, greater metabolic rate, higher heat production and poor heat resistance due to selective breeding (Deeb and Cahaner, 2002; Settar et al., 1999). Therefore, broilers are susceptible to environmental hyperthermia and heat stress (HS) can easily cause poor growth performance and poor health status of broilers (Schirmann et al., 2021). The global average surface temperature continues to rise in recent years (Ma et al., 2022a), thus the poultry industry, with high-density intensive production, is exposed to the enormous challenge of HS. HS induced-poor growth performance of broilers is generally accompanied by skeletal muscle growth retardation and poor meat quality (Liu et al., 2022; Ma et al., 2018; Wen et al., 2019). Current evidence shows that HS causes mitochondrial stress, suppresses the activity of the mitochondrial respiratory chain, and increases the production of reactive oxygen species (ROS), which ultimately induces oxidative damage (Tan et al., 2010). HS-induced oxidative damage in skeletal muscle is mainly reflected in two aspects: inhibition of protein biosynthesis and activation of protein degradation (Gao et al., 2015; Ma et al., 2018). Excessive ROS inhibits protein biosynthesis mainly though suppressing the mammalian target of rapamycin (mTOR) pathway (Toshniwal et al., 2019). Besides, HS-mediated mitochondrial stress activates apoptotic signaling in skeletal muscle, which ultimately facilitates protein degradation (Ganesan et al., 2016). Hence, mitochondrial stress may be the key factor that mediates HS-induced skeletal muscle growth retardation and poor meat quality in broilers.

The rough endoplasmic reticulum (ER) is the key organelle for protein processing and transportation. Under stress conditions, excessive ROS impairs the ER homeostasis, causing ER stress, which impairs the efficiency of protein assembly and transportation (Han et al., 2013). ER stress generally activates the caspase and ubiquitin proteasome system, thus promoting protein degradation and impairing the normal growth of skeletal muscle (Li et al., 2016). When ER homeostasis is disrupted, excessive unfolded proteins in the ER lumen activate the ER unfolded protein response (ER-UPR) to mitigate ER stress, thus the ER-UPR appears to be representative of ER stress (Lemmer et al., 2021). The activation of ER-UPR is generally through three ER transmembrane sensors: protein kinase-like endoplasmic reticulum kinase (PERK), inositol-requiring protein 1 (IRE1), and activating transcription factor 6 (ATF6) (Calfon et al., 2002; Carrara et al., 2015; Ye et al., 2000). Evidence suggests that activation of ER-UPR causes the accumulation of hypophosphorylated (active) eukaryotic translation initiation factor 4E (eIF4E) binding proteins, thus suppressing mRNA translation (Hamanaka et al., 2006). Hence, it is reasonable to expect that alleviation of mitochondrial dysfunction and ER stress may be an effective measure to relieve HS-induced skeletal muscle growth retardation and poor meat quality of broilers.

As an essential trace element for animals, selenium (Se) is involved in the composition of the antioxidant system and plays a unique role in ROS removal and redox maintenance (Rayman, 2012; Tang et al., 2018). Se deficiency induces multiple tissue damage with dysregulation of redox homeostasis in broilers under HS (Zhao et al., 2023). Previous studies posit that Se supplementation can maintain mitochondrial and ER homeostasis of cells and organs to ensure normal metabolism (Jing et al., 2022; Liu et al., 2017). Se exerts its biological functions mainly though selenoproteins of which there are 24 that have been found in broilers. Among these selenoproteins, three of them (glutathione peroxidase 4 [GPX4], selenoprotein O [SELENOO] and thioredoxin reductase 2 [TXNRD2]) are identified as mitochondrial selenoproteins and seven (deiodinase 2 [DIO2], selenoprotein F [SELENOF], selenoprotein K [SELENOK], selenoprotein M [SELENOM], selenoprotein N [SELENON], selenoprotein S [SELENOS] and selenoprotein T [SELENOT]) are located in the ER (Reeves and Hoffmann, 2009). These mitochondrial or ER resident selenoproteins play irreplaceable roles in mitochondrial and ER homeostasis maintenance. Further, free selenoproteins such as glutathione peroxidase 1 (GPX1), glutathione peroxidase 2 (GPX2), glutathione peroxidase 3 (GPX3) and selenoprotein W (SELENOW) could work closely with resident selenoproteins in organelles to clear excessive ROS and recover cell homeostasis (Jing et al., 2022; Liu et al., 2021; Tang et al., 2019). Generally, the expression of selenoproteins in animal muscle can be effectively regulated by dietary Se levels (Sunde and Raines, 2011), and organic Se can linearly increase the Se concentration in muscle (Liu et al., 2021; Tang et al., 2021). Therefore, our present study focuses on the impact of chronic heat stress (CHS) on skeletal muscle growth, meat quality, mitochondrial dysfunction and ER stress, and the potential mechanism linked to the protective effects of organic Se in the form of selenomethionine (SeMet).

## 2 Materials and methods

### 2.1 Animal ethics statement

The animal trial was approved by the Animal Care and Use Committee of the Laboratory Animal Center at Sichuan Agricultural University (Ethics Approval Code: SCAUAC202107-1).

### 2.2 Animals, diets and experimental design

In this study, 500 Arbor Acres male broilers aged 1 d with average body weight of 47 ± 5 g were randomly grouped into 5 treatments with 10 replicates of 10 broilers per replicate. Broilers in 1 treatment (control group, CON) were fed a basal diet (formulated in accordance with Nutrient Requirements of Poultry (NRC, 1994), details in Table S1). The other 4 treatments were fed the basal diet with 0.0, 0.2, 0.4 and 0.6 mg Se/kg SeMet (Se concentration in SeMet is 2000 mg/kg, and the determined dietary Se concentration is shown in Fig. S1A), pretreatment for 21 d. After 21 d, 80 broilers in each treatment with average body weight of 680 ± 70 g were selected and divided into 8 replicates and 10 broilers per replicate for formal testing. Broilers in the control group were raised in a thermoneutral environment (22 ± 2 °C) and fed on the basal diet; the other 4 treatments were exposed to hyperthermic condition (33 ± 2 °C, 24 h in each day) and fed on the basal diet (CHS) or basal diet with 0.2 (CHS + 0.2 Se), 0.4 (CHS + 0.4 Se) and 0.6 (CHS + 0.6 Se) mg Se/kg SeMet (determined dietary Se concentration is shown in Fig. S1B). The formal experiment lasted for 21 d.

Broilers in each replicate were housed in a cage of the same size (length × width × height, 100 cm × 80 cm × 60 cm). Broilers in the control group were kept in a separate house (the construction and equipment of the two houses used for the control group and treatment groups were consistent). The poultry farm monitoring system (ROTEM AC-2000 PLUS, AgroLogic Ltd., Israel) was used to control the temperature and relative humidity. The extra heat for the CHS group was provided by an electric hot air blower (50 KW, Shandong Machinery Co., Ltd., Qingzhou, China). The extra relative humidity in the CHS group was regulated by a fog machine (PC-2804, Taizhou Paichi Machinery Co., Ltd., Taizhou, China). For the 21 d pretreatment period, broilers in each treatment were in the same temperature and relative humidity, then the temperature was gradually decreased from 33 to 22 °C and the relative humidity kept at 50%. For the 21 d formal test period, during the first 6 d, the temperature and relative humidity in the CHS groups were gradually increased from 22 °C and 50% to 33 °C and 65%, respectively. Then, the temperature was kept at 33 ± 2 °C and the relative humidity was kept at 65% ± 5% in the CHS groups, while the temperature was kept at 22 ± 2 °C and the relative humidity was kept at 50% ± 5% in the CON group, until the end of the trial. Broilers had free access to diet and water and the temperature and relative humidity were monitored and recorded every day.

### 2.3 Feed sample collection and chemical analyses

Diets were formulated twice during the experiment; each batch of diet samples (200 g) from different treatment groups was collected and kept at −20 °C for lab analysis. The nutrient composition (crude protein, total Ca, total P) of diets was analyzed (3 replicates for each treatment group) according to the method described in Association of Official Analytical Chemists (AOAC, 2005). The dry matter of feed was determined by drying in an oven at 105 °C until a constant weight. The crude protein in feed was analyzed using the automatic Kjeldahl nitrogen analyzer (Kjeltec 8420, FOSS, Denmark) and calculated using the factor 6.25 for nitrogen. The total calcium and phosphorus in feed were determined using a graphite furnace atomic absorption spectrometer (novAA400, Analytik Jena AG, Germany). ME was calculated according to Nutrient Requirements of Poultry (NRC, 1994).

### 2.4 Growth performance and respiration rate of broilers

The body weight and feed intake were determined at the beginning and the end of the trial. The average daily gain (ADG), average daily feed intake (ADFI) and feed conversion ratio (FCR) were calculated as follows: ADG = [total body gain (g)/test days]/number of broilers in each replicate, ADFI = [total feed intake (g)/test days]/number of broilers in each replicate, FCR = ADFI/ADG. The respiration rate of broilers was recorded on d 21, 28, 35 and 42, respectively. A manual mechanical counter was used to determine the respiration rate; one broiler chicken in each replicate (a total of 40 broilers) was tracked for 30 s, then the rate was quantified and expressed as breaths per minute.

### 2.5 Breast muscle weight and sample collection

At the end of the experiment, a total of 30 broilers (6 broilers in each group with a body weight close to the average body weight) were selected. After an overnight fasting, the broilers were weighed and euthanized by cervical dislocation. Bilateral breast muscles were weighed after being completely detached. A small part of the right breast muscle was cut and collected in a sterile tube, then snap-frozen in liquid nitrogen and stored at −80 °C for Se concentration, enzyme activity, RT-PCR and Western blot detection.

### 2.6 Meat quality of breast muscle

#### 2.6.1 Meat color and pH

The left breast muscle was cut into a large and a small piece, the large piece was used to determine the meat color and pH according to a previous report (Honikel, 1998). Meat color attributes including lightness (*L∗*), redness/greenness (*a∗*) and yellowness/blueness (*b∗*) values, at 45 min and 24 h were determined using a Minolta Chromameter CR-300 (Minolta Camera, Osaka, Japan). The 45 min and 24 h pH values were measured using a portable pH meter (pH-Star, Matthäus, Pöttmes, Germany). Three different points in each breast muscle sample were selected to determine meat color and pH; the calculated mean values were used for statistical analysis.

#### 2.6.2 Cooking loss

The small piece of the left breast muscle samples was used to determine the cooking loss according to a previous method (Honikel, 1998). In brief, the meat samples were stored at 4 °C for 24 h, then about 30 g of samples were weighed at room temperature and recorded as *W1*. The samples were put into a plastic bag and cooked to an internal temperature of 70 °C in an 80 °C water bath. Internal temperature was monitored during cooking with a kerosene thermometer. Then, the meat sample was taken out and cooled for 60 min, blotted dry and weighed as *W2*. Cooking loss (%) = (*W1* − *W2*)/*W1* × 100.

#### 2.6.3 Drip loss

The drip loss was determined according to a previous report (Honikel, 1998). Briefly, a fresh 5 cm × 3 cm × 0.5 cm thick slice from each right breast muscle was weighed and recorded as *W3*. Each sample was put into a plastic bag and suspended for 24 h and 48 h in a 4 °C cooler, then taken out and reweighed as *W4* and *W5*, respectively. The 24 h drip loss (%) = (*W3* − *W4*)/*W3* × 100; 48 h drip loss (%) = (*W3* − *W5*)/*W3* × 100.

#### 2.6.4 Peak shear force

After measuring the 24 h meat color and pH, the large piece of the left breast muscle samples was used to determine peak shear force according to a previous method (Honikel, 1998). The samples were placed into a plastic bag and cooked to an internal temperature of 70 °C in a 90 °C water bath. Internal temperature was monitored during cooking with a kerosene thermometer. Then, all breast muscle samples were taken out and cooled to room temperature and stored at 4 °C for 24 h. Five cores (1.2 cm diameter) from each sample were taken parallel to muscle fiber direction with the same sampler. Each core was sheared using the Texture Analyzer (XT2, Stable Micro Systems Ltd., Godalming, Surrey, UK), and peak shear force was recorded; the calculated mean values were used for statistical analysis.

### 2.7 Selenium concentration in diets and breast muscle

The concentration of Se in diets and breast muscle was measured by using a hybrid generation-atomic fluorescence spectrometer (AFS-230E, Beijing Haiguang instrument, China) against the standard Se reference (China National Standard, 2008; 2010; National Research Centre for Certified Reference Materials, Beijing, China). Sample pretreatment has been described previously (Tang et al., 2021).

### 2.8 Antioxidant capacity and enzyme analyses

The activity of total glutathione peroxidase (GSH-Px), total antioxidant capability (T-AOC) and total superoxide dismutase (T-SOD) in breast muscle were measured by using the corresponding assay kits (no. A005, A015-1, A001-1-1, Nanjing Jiancheng Bioengineering Institute, Nanjing, China). The concentration of malondialdehyde (MDA) in breast muscle was determined using a commercial kit (no. A003-1, Nanjing Jiancheng Bioengineering Institute, Nanjing, China). The activity of ubiquitin-activating enzyme E1 (UBE1) in breast muscle was determined using a commercial enzyme-linked immunosorbent assay (ELISA) kit (no. MM-6068702, Jiangsu Meimian Industrial Co., Ltd., Jiangsu, China). The protein levels in each sample were determined with the bicinchoninic acid (BCA) method by using a commercial assay kit (no. A045-3, Nanjing Jiancheng Bioengineering Institute, Nanjing, China). The optical density values were measured with an ultraviolet–visible spectrophotometer (Model 680, Bio-Rad, CA, USA).

### 2.9 ATP and ROS levels in breast muscle

The ATP levels in each breast muscle sample were determined by using a commercial assay kit (no. A095-1-1, Nanjing Jiancheng Bioengineering Institute, Nanjing, China) according to the manufacturer's instructions. The concentration of ROS in breast muscle was measured using an ELISA kit (no. MM-6012001, Jiangsu Meimian Industrial Co., Ltd., Jiangsu, China) according to the manufacturer's instructions.

### 2.10 RT-PCR analysis of mRNA expression in breast muscle

As used previously (Zhao et al., 2015), the total RNA in each breast muscle sample was extracted by using RNAiso Plus (no. 9109, Takara, Dalian, China). After inspection by nucleic acid electrophoresis, 3000 ng total RNA were used for cDNA synthesis with the ExonScript RT SuperMix kit (no. A502-02, EXONGEN, Chengdu, China). The qPCR was performed in a final volume of 10 μL using a Fast SYBR Green qPCR Master Mix UDG kit (no. A402-1, EXONGEN, Chengdu, China) on the QuantStudio 5 Flex system (Applied Biosystems, CA, USA). The relative mRNA expression was normalized to the expression of β-actin and calculated by using the 2^−ΔΔCt^ method. The primers for all the tested genes and β-actin were designed using Primer Express 3.0 (Applied Biosystems, CA, USA), and listed in Table S2.

### 2.11 Western blot analysis of protein abundance in breast muscle

Western blot was performed as described previously (Liu et al., 2022). In brief, the breast muscle samples were homogenized in ice-cold radio immunoprecipitation assay (RIPA) buffer with phenylmethanesulfonyl fluoride (PMSF) protease inhibitor buffer (Beyotime, Shanghai, China), and centrifuged (13,000 × *g*) for 30 min at 4 °C. Then, the supernatant of each sample was collected and the total protein concentration was measured using the BCA kit (no. A045-3, Nanjing Jiancheng Bioengineering Institute, Nanjing, China). The protein concentration of each sample was adjusted to 6 μg/μL, then 400 μL of the above protein samples were mixed with 100 μL SDS-PAGE sample loading buffer (5×) (no. P0015L, Beyotime, Shanghai, China) and denatured in a 95 °C water bath for 10 min. The protein samples (8 μL) were separated by using 8% to 12% SDS-PAGE gels and transferred onto polyvinylidene difluoride membranes (Bio-Rad, CA, USA). The above membranes were blocked with 5% defatted milk for 2 h and washed 3 times with Tris buffered saline with Tween 20 (TBST) (prepared with Tris–HCl buffer and isotonic salt solution containing 1% Tween 20) and incubated overnight at 4 °C with primary antibodies (detailed in Table S3). Then, these membranes were incubated with corresponding secondary antibodies (anti-rabbit or anti-mouse IgG, 1:5000; Proteintech Group, IL, USA). The bands were visualized by using an enhanced chemiluminescence system (Bio-Rad, CA, USA), and quantified using the Image Lab software system (Bio-Rad, CA, USA).

### 2.12 Statistical analysis

The data of mortality was represented as a percentage and the nonparametric chi-square test was performed with SPSS 27.0 (SPSS Inc., Chicago, USA) to analyze the mortality. Other data are expressed as means with standard error of the mean (SEM) or standard errors. Statistical analyses were performed with SPSS 27.0 (SPSS Inc., Chicago, USA), values were analyzed using one-way ANOVA followed by Tukey's multiple range tests, and ANOVA *P*-values of less than 0.05 were considered statistically significant. Principal component analysis of selenotranscriptome in breast muscle was accomplished by SPSS 27.0 (SPSS, Inc., Chicago, USA). All results were plotted using GraphPad Prism Version 8 software (GraphPad Software, LLC, San Diego, USA).

## 3 Results

### 3.1 Respiration rate and growth performance of broilers

**Fig. 1:**
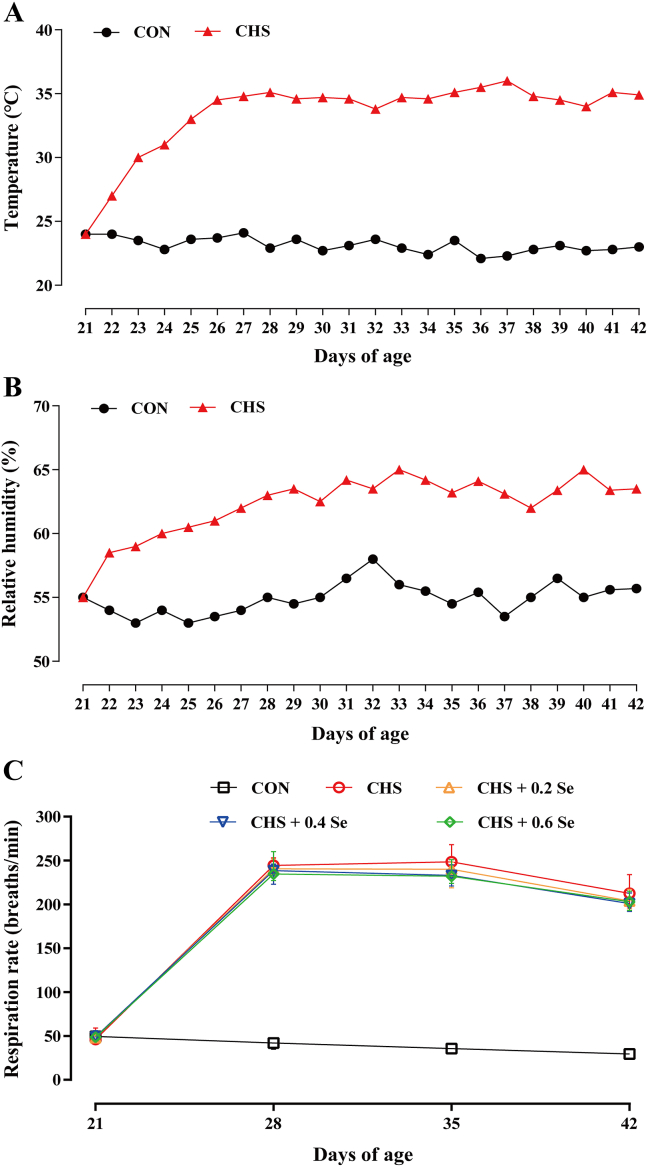
Ambient temperature, room relative humidity, and respiration rate of broilers. (A) Ambient temperature during the experiment. (B) Room relative humidity during the experiment. (C) Respiration rate of broilers. CON = control group; CHS = chronic heat stress group; CHS + 0.2 Se = chronic heat stress + 0.2 Se mg/kg selenomethionine (SeMet) group; CHS + 0.4 Se = chronic heat stress + 0.4 Se mg/kg SeMet group; CHS + 0.6 Se = chronic heat stress + 0.6 Se mg/kg SeMet group. Results for respiration rate was expressed as mean ± SD (*n* = 8).

The temperature and relative humidity were recorded daily during the trial. Compared with the CON group, CHS sharply increased the respiration rate (Fig. 1C) and lead to growth retardation of broilers. After exposure to CHS for 21 d, the body weight of broilers decreased (*P* < 0.05) by 29.5% (Table 1). CHS decreased (*P* < 0.05) the ADFI and ADG, increased the FCR (*P* < 0.05) of broilers (Table 1). Dietary SeMet supplementation, especially 0.4 and 0.6 mg Se/kg, partly alleviated the negative effects of CHS, which increased the final body weight and lowered the FCR of broilers (Table 1). No dead broilers were found in the CON group, while the mortality of the CHS group was 2.50%. SeMet supplementation exhibited limited impact on the mortality of broilers exposed CHS.

**Table 1:** Effects of chronic heat stress and selenomethionine (SeMet) supplementation on growth performance of broilers.

Item	CON	CHS	CHS +	CHS +	CHS +	SEM/χ^2^	*P*-value
			0.2 Se	0.4 Se	0.6 Se		
1–21 d							
BW, g/bird							
1 d	47.0	46.9	46.9	47.0	47.1	<0.01	0.900
21 d	677.6	674.5	675.6	692.5	684.7	0.04	0.569
ADFI, g/d	47.38	47.74	47.79	48.43	47.47	0.223	0.610
ADG, g/d	30.03	29.89	30.24	30.74	30.36	0.186	0.635
FCR	1.58	1.60	1.58	1.58	1.56	0.009	0.837
22–42 d							
BW, g/bird							
22 d	684.1	683.0	683.9	684.5	682.8	0.31	0.325
42 d	2266.8^a^	1598.9^c^	1641.5^bc^	1657.1^b^	1633.6^bc^	41.28	<0.001
ADFI, g/d	139.16^a^	92.62^b^	94.60^b^	94.59^b^	92.74^b^	2.995	<0.001
ADG, g/d	75.36^a^	43.62^b^	45.60^b^	46.51^b^	45.28^b^	1.962	<0.001
FCR	1.85^c^	2.13^a^	2.08^ab^	2.04^b^	2.05^b^	0.016	<0.001
Mortality, %	0.00	2.50	1.25	2.50	1.25	χ^2^ = 2.369	0.668

BW = body weight; ADFI = average daily feed intake; ADG = average daily gain; FCR = feed conversion rate. CON = control group; CHS = chronic heat stress group; CHS + 0.2 Se = chronic heat stress + 0.2 Se mg/kg selenomethionine (SeMet) group; CHS + 0.4 Se = chronic heat stress + 0.4 Se mg/kg SeMet group; CHS + 0.6 Se = chronic heat stress + 0.6 Se mg/kg SeMet group. Results for BW, ADFI, ADG and FCR were expressed as mean with SEM (*n* = 8). ^a-c^Different letters indicate significant differences (*P* < 0.05).

### 3.2 Dysplasia of breast muscle and meat quality

**Fig. 2:**
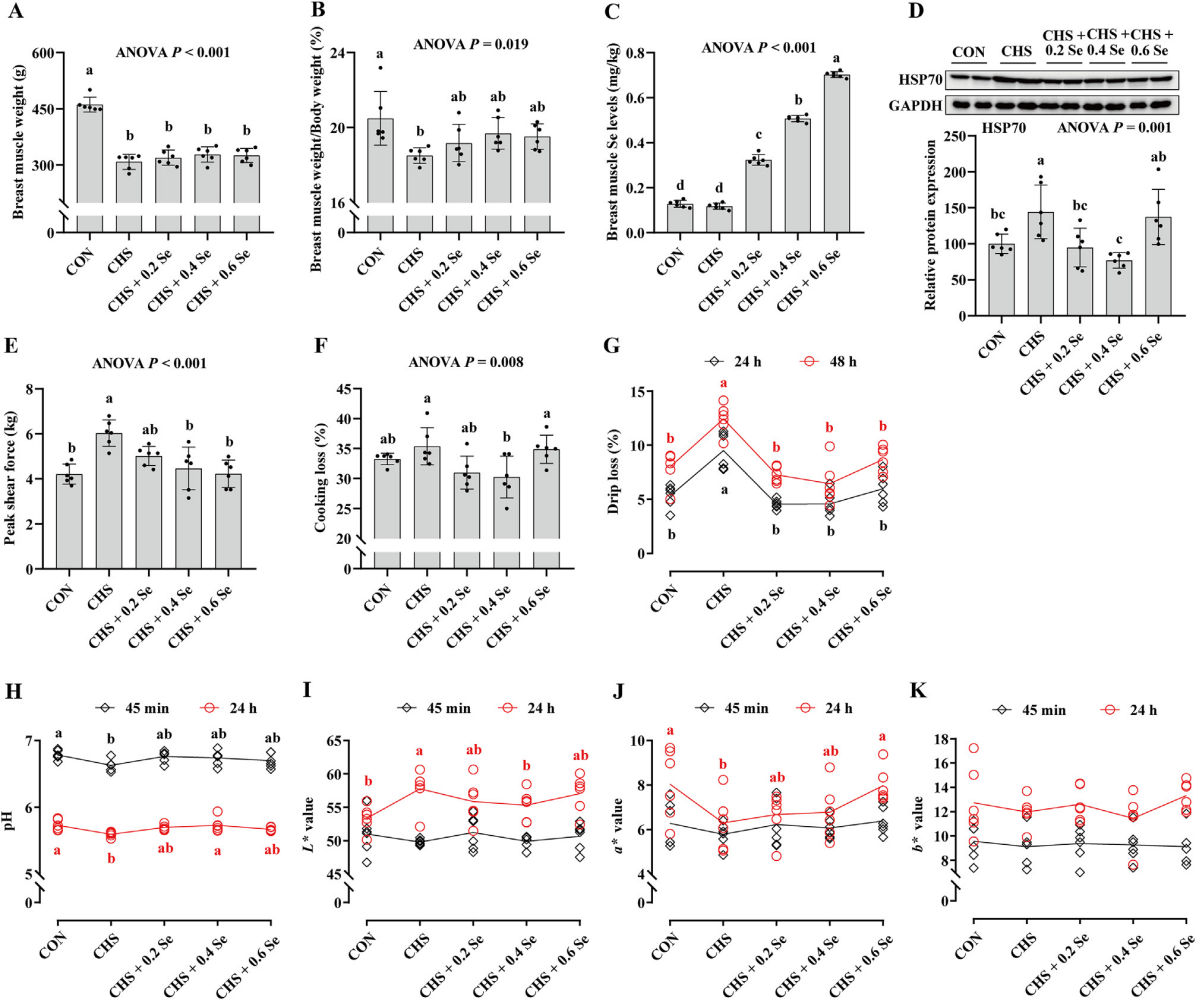
Dysplasia of breast muscle and meat quality. (A) Breast muscle weight of broilers. (B) Breast muscle percentage of broilers. (C) Breast muscle Se deposition of broilers. (D) Protein expression of HSP70 in breast muscle of broilers. (E) The peak shear force of breast muscle. (F) Cooking loss of breast muscle. (G) Drip loss of breast muscle. (H) The pH value of breast muscle. (I) The *L∗* value of breast muscle. (J) The *a∗* value of breast muscle. (K) The *b∗* value of breast muscle. HSP70 = heat shock protein 70; *L∗* = lightness; *a∗* = redness/greenness; *b∗* = yellowness/blueness. CON = control group; CHS = chronic heat stress group; CHS + 0.2 Se = chronic heat stress + 0.2 Se mg/kg selenomethionine (SeMet) group; CHS + 0.4 Se = chronic heat stress + 0.4 Se mg/kg SeMet group; CHS + 0.6 Se = chronic heat stress + 0.6 Se mg/kg SeMet group. Results were expressed as mean ± SD (for breast muscle weight, breast muscle percentage, protein expression of HSP70, peak shear force, cooking loss, drip loss, pH value, *L∗* value, *a∗* value, and *b∗* value, the *n* = 6; for breast muscle Se deposition, the *n* = 4). ^a-d^Different letters indicate significant differences (*P* < 0.05).

Compared with the CON group, broilers exposed to CHS yielded lower (*P <* 0.05) breast muscle weight (Fig. 2A) and breast muscle index (Fig. 2B). Besides, CHS group up-regulated (*P <* 0.05) the protein abundance of heat shock protein 70 (HSP70) (Fig. 2D). Dietary Se supplementation exhibited protective effects, which linearly increased (*P <* 0.05) the Se concentration in breast muscle (Fig. 2C), decreased (*P <* 0.05) the protein expression of HSP70 (Fig. 2D), and increased (0.05 < *P* < 0.10) the breast muscle index to a certain extent (Fig. 2B).

Furthermore, CHS caused poor meat quality of breast muscle, which increased (*P <* 0.05) the peak shear force, cooking loss, drip loss and 24 h *L∗* value, while decreasing (*P <* 0.05) pH and 24 h *a∗* value (Fig. 2E–J). Dietary Se supplementation improved the meat quality of broilers under CHS, which decreased the peak shear force, drip loss and 24 h *L∗* value (Fig. 2E–G and I). Besides, 0.4 mg Se/kg SeMet decreased (*P <* 0.05) the cooking loss (Fig. 2F), and Se supplementation increased the pH and 24 h *a∗* value (Fig. 2H and J).

### 3.3 Apoptosis, ubiquitination and mTOR pathway in breast muscle

**Fig. 3:**
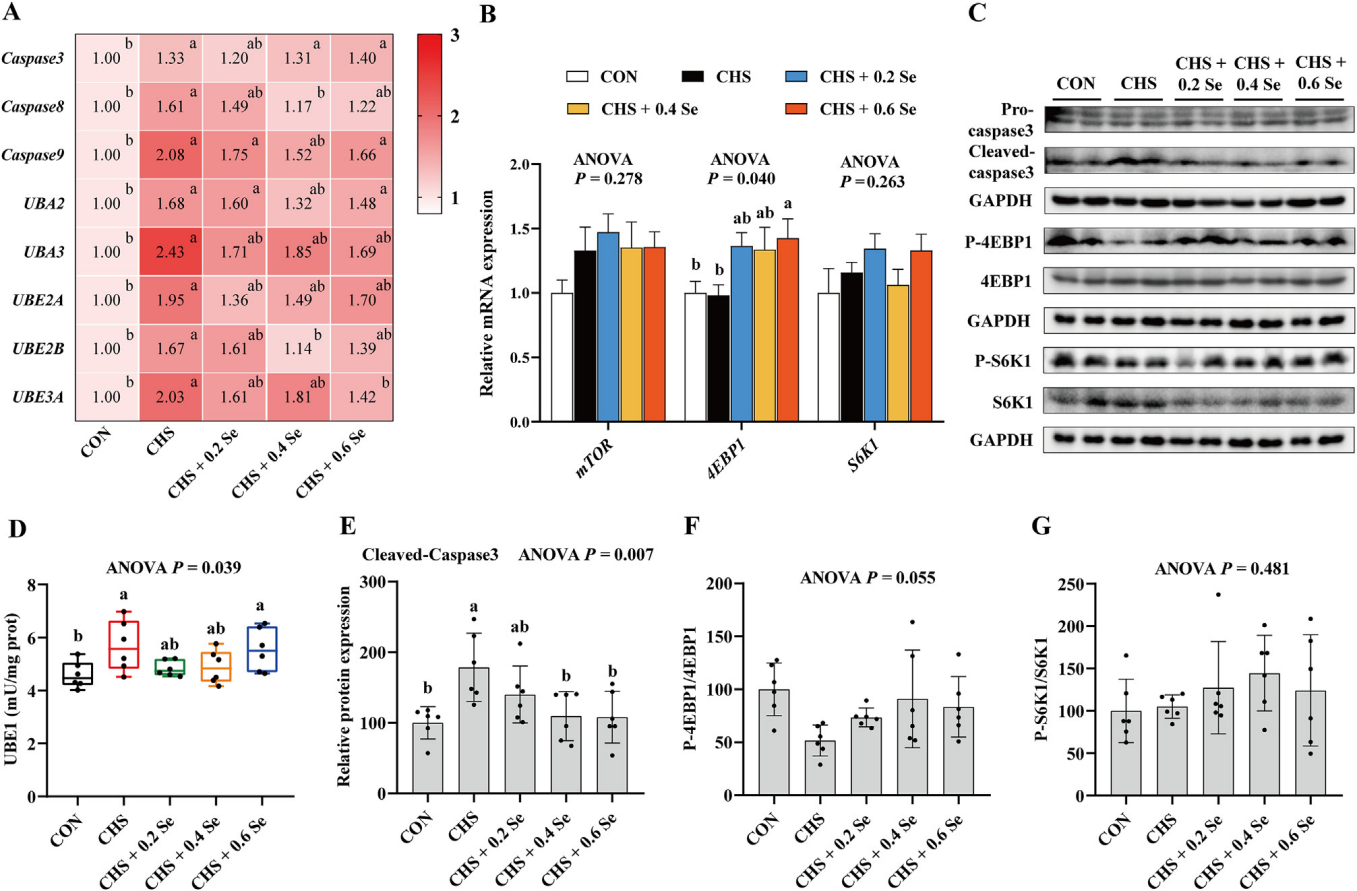
Apoptosis, ubiquitination and mTOR pathway in breast muscle. (A) Relative mRNA expression of caspase 3, caspase 8, caspase 9, *UBA2*, *UBA3*, *UBE2A*, *UBE2B* and *UBE3A* in breast muscle. (B) Relative mRNA expression of *mTOR*, *4EBP1* and *S6K1* in breast muscle. (C) Protein abundance of caspase 3, 4EBP1 and S6K1 in breast muscle. (D) Activity of UBE1 in breast muscle. (E) Relative protein expression of cleaved-caspase 3 in breast muscle. (F) Relative protein expression of P-4EBP1 in breast muscle. (G) Relative protein expression of P-S6K1 in breast muscle. mTOR = mammalian target of rapamycin; 4EBP1 = eukaryotic translation initiation factor 4E (eIF4E)-binding protein 1; S6K1 = mitogen-stimulated protein kinase p70 ribosomal protein S6 kinase 1; *UBA2* = ubiquitin-like modifier activating enzyme 2; *UBA3* = ubiquitin-like modifier activating enzyme 3; *UBE2A* = ubiquitin conjugating enzyme E2 A; *UBE2B* = ubiquitin conjugating enzyme E2 B; *UBE3A* = ubiquitin protein ligase E3 A. CON = control group; CHS = chronic heat stress group; CHS + 0.2 Se = chronic heat stress + 0.2 Se mg/kg selenomethionine (SeMet) group; CHS + 0.4 Se = chronic heat stress + 0.4 Se mg/kg SeMet group; CHS + 0.6 Se = chronic heat stress + 0.6 Se mg/kg SeMet group. Results were expressed as mean ± SD (*n* = 6). ^a,b^Different letters indicate significant differences (*P* < 0.05).

Compared with the CON group, CHS group up-regulated (*P <* 0.05) the mRNA expression of caspase 3, caspase 8, caspase 9, ubiquitin-like modifier activating enzyme 2 (*UBA2*), ubiquitin-like modifier activating enzyme 3 (*UBA3*), ubiquitin conjugating enzyme E2 A (*UBE2A*), ubiquitin conjugating enzyme E2 B (*UBE2B*) and ubiquitin protein ligase E3 A (*UBE3A*) (Fig. 3A). CHS also increased (*P <* 0.05) the activity of UBE1 (Fig. 3D) and the protein abundance of cleaved-caspase 3 (Fig. 3C and E). Compared with the CHS group, broilers that received Se showed lower mRNA levels of these 7 genes (caspase 8, caspase 9, *UBA2*, *UBA3*, *UBE2A*, *UBE2B* and *UBE3A*), especially 0.4 mg Se/kg, which significantly decreased (*P <* 0.05) the mRNA levels of caspase 8 and *UBE2B*, and 0.6 mg Se/kg decreased (*P <* 0.05) the mRNA levels of *UBE3A*. Further, 0.2 and 0.4 mg Se/kg SeMet decreased (0.05 < *P* < 0.10) the UBE1 activity to a certain extent, 0.4 and 0.6 mg Se/kg SeMet decreased (*P* < 0.05) the protein levels of cleaved-caspase 3.

Compared with the CON group, CHS group exhibited limited impacts (*P >* 0.05) on the mRNA levels of *mTOR*, eIF4E-binding protein 1 (*4EBP1*) and mitogen-stimulated protein kinase p70 ribosomal protein S6 kinase 1 (*S6K1)* (Fig. 3B), while tending to decrease (*P* = 0.055) the protein level of P-4EBP1 (Fig. 3C and F). Dietary Se supplementation up-regulated the mRNA levels of *4EBP1* and increased (0.05 < *P* < 0.10) the protein levels of P-4EBP1. However, these changes did not show dose-dependence.

### 3.4 Antioxidant status of breast muscle

**Fig. 4:**
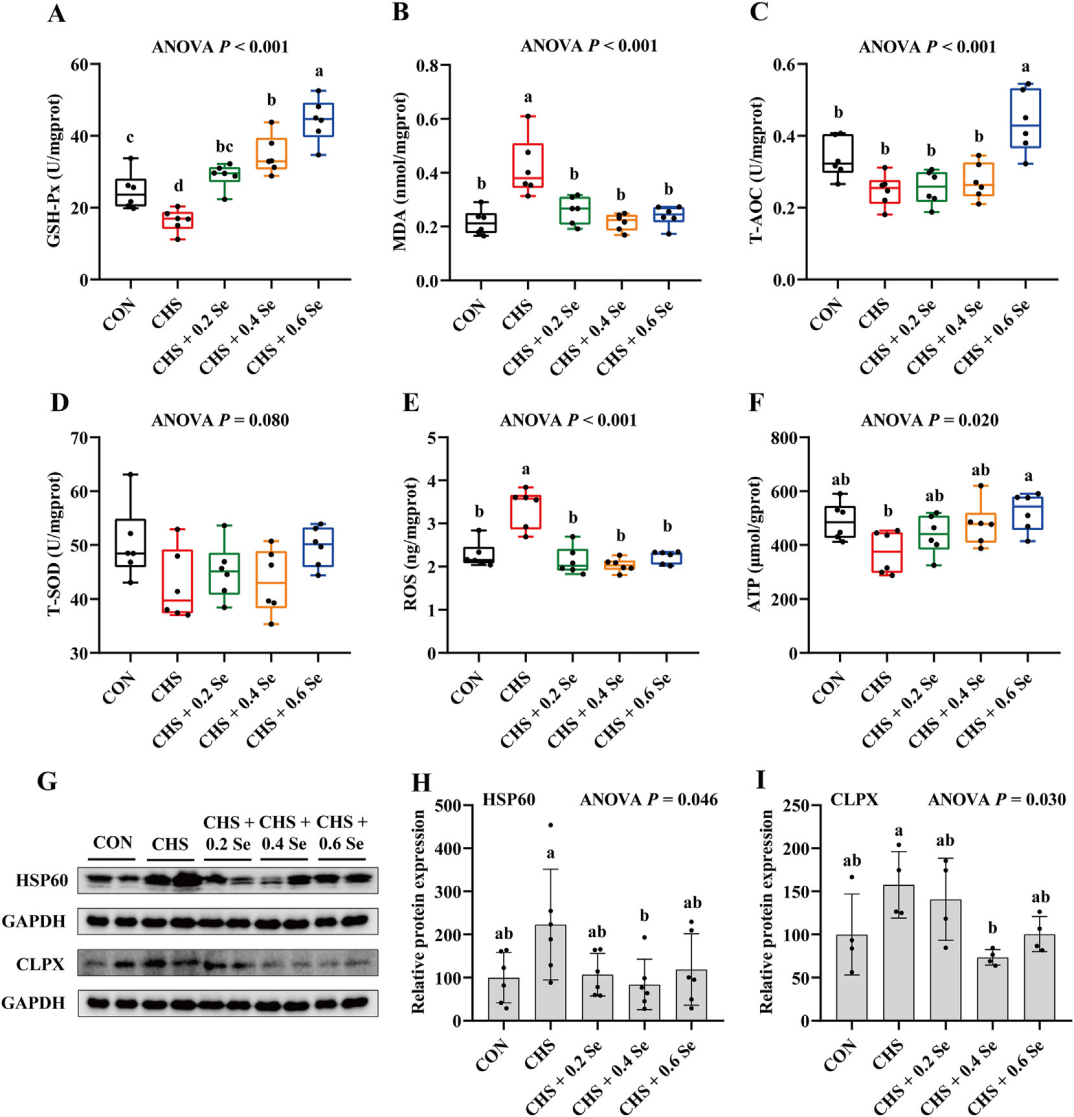
Antioxidant status and mitochondrial dysfunction biomarkers in breast muscle. (A) Activity of total GSH-Px in breast muscle. (B) MDA concentration in breast muscle. (C) Activity of T-AOC in breast muscle. (D) Activity of T-SOD in breast muscle. (E) ROS concentration in breast muscle. (F) ATP concentration in breast muscle. (G) Relative protein expression of HSP60 and CLPX in breast muscle. (H) Relative protein expression of HSP60. (I) Relative protein expression of CLPX. GSH-Px = glutathione peroxidase; MDA = malondialdehyde; T-AOC = total antioxidant capability; T-SOD = total superoxide dismutase; ROS = reactive oxygen species; ATP = adenosine triphosphate; HSP60 = heat shock protein 60; CLPX = ATP-dependent CLP protease ATP-binding subunit. CON = control group; CHS = chronic heat stress group; CHS + 0.2 Se = chronic heat stress + 0.2 Se mg/kg selenomethionine (SeMet) group; CHS + 0.4 Se = chronic heat stress + 0.4 Se mg/kg SeMet group; CHS + 0.6 Se = chronic heat stress + 0.6 Se mg/kg SeMet group. Results were expressed as mean ± SD (for GSH-Px activity, MDA concentration, T-AOC activity, T-SOD activity, ROS concentration, ATP concentration, protein expression of HSP60, the *n* = 6; for the protein expression of CLPX, the *n* = 4). ^a-d^Different letters indicate significant differences (*P* < 0.05).

The antioxidant status of breast muscle was determined. Compared with the CON group, CHS group decreased the antioxidant capacity of breast muscle, which lowered (*P <* 0.05) the activity of GSH-Px (Fig. 4A) and increased (*P* < 0.05) the MDA levels (Fig. 4B). Dietary Se supplementation, especially 0.6 mg Se/kg, improved the antioxidant capacity, which increased the activity of GSH-Px (Fig. 4A) and T-AOC (Fig. 4C), and decreased the MDA levels (Fig. 4B), compared with the CHS group.

### 3.5 Mitochondrial homeostasis in breast muscle

To evaluate whether CHS disrupts mitochondrial homeostasis and the protective effects of SeMet, the levels of ATP, ROS and protein abundance of heat shock protein 60 (HSP60) and ATP-dependent CLP protease ATP-binding subunit (CLPX) were determined. Compared with the CON group, broilers faced with CHS showed a higher (*P <* 0.05) ROS level (Fig. 4E) and a lower (0.05 < *P* < 0.10) ATP level (Fig. 4F) in breast muscle. In addition, CHS tended to increase (0.05 < *P* < 0.10) the protein abundance of HSP60 and CLPX (Fig. 4G–I). Dietary Se supplementation relieved the negative effects of CHS, which decreased (*P* < 0.05) the ROS levels, increased the ATP levels and decreased the protein abundance of HSP60 and CLPX.

### 3.6 Endoplasmic reticulum homeostasis in breast muscle

**Fig. 5:**
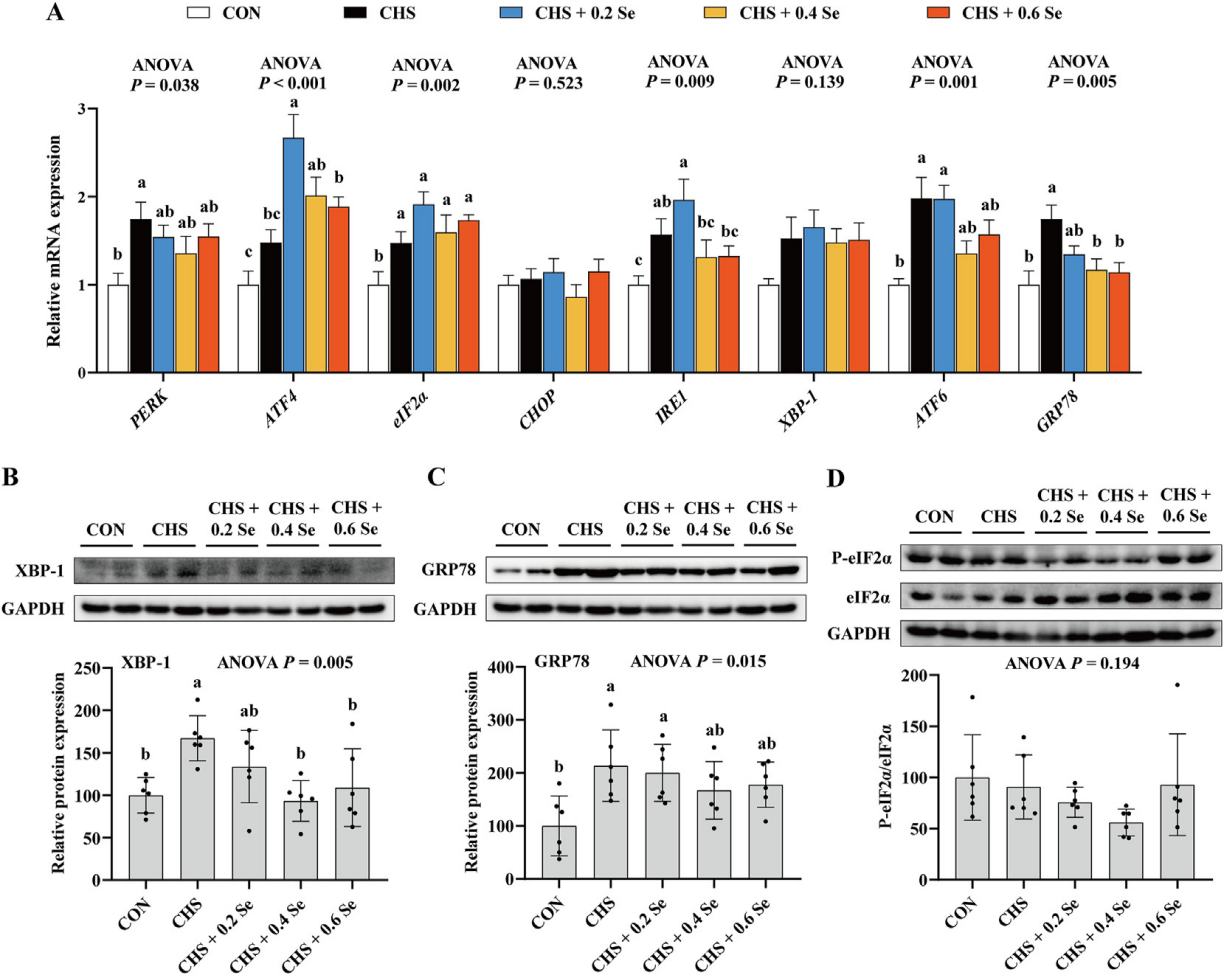
Endoplasmic reticulum (ER) stress biomarkers in breast muscle. (A) Relative mRNA expression of ER stress biomarkers in breast muscle. (B) Relative protein expression of XBP-1 in breast muscle. (C) Relative protein expression of GRP78 in breast muscle. (D) Relative protein expression of P-eIF2α in breast muscle. *PERK* = protein kinase-like endoplasmic reticulum kinase; *ATF4* = activating transcription factor 4; eIF2α = eukaryotic initiation factor-2α; *CHOP* = C/EBP homologous protein; *IRE1* = inositol-requiring protein 1; *XBP-1* = X-box binding protein 1; *ATF6* = activating transcription factor 6; GRP78 = glucose regulatory protein 78. CON = control group; CHS = chronic heat stress group; CHS + 0.2 Se = chronic heat stress + 0.2 Se mg/kg selenomethionine (SeMet) group; CHS + 0.4 Se = chronic heat stress + 0.4 Se mg/kg SeMet group; CHS + 0.6 Se = chronic heat stress + 0.6 Se mg/kg SeMet group. Results were expressed as mean ± SD (*n* = 6). ^a-c^Different letters indicate significant differences (*P* < 0.05).

We further determined the ER stress biomarkers in breast muscle of broilers. Compared with the CON group, CHS group up-regulated (*P <* 0.05) the mRNA expression of *PERK*, eukaryotic initiation factor-2α (*eIF2α*), *IRE1*, *ATF6* and glucose regulatory protein 78 (*GRP78*) (Fig. 5A), and increased (*P <* 0.05) the protein abundance of X-box binding protein 1 (XBP-1) (Fig. 5B) and GRP78 (Fig. 5C). Dietary Se supplementation exhibited positive effects on these ER stress biomarkers. It was revealed that 0.4 and 0.6 mg Se/kg SeMet decreased (*P <* 0.05) the mRNA expression of *GRP78* and tended to decrease (0.05 < *P* < 0.10) the mRNA levels of *PERK*, *IRE1* and *ATF6* (Fig. 5A). Further, 0.4 and 0.6 mg Se/kg SeMet decreased (*P <* 0.05) the protein abundance of XBP-1 (Fig. 5B), and tended to decrease (0.05 < *P* < 0.10) the protein level of GRP78 (Fig. 5C). CHS and Se supplementation exhibited limited impact on the protein abundance of P-eIF2α (Fig. 5D).

### 3.7 Expression of selenotranscriptome and key selenoproteins in breast muscle

**Fig. 6:**
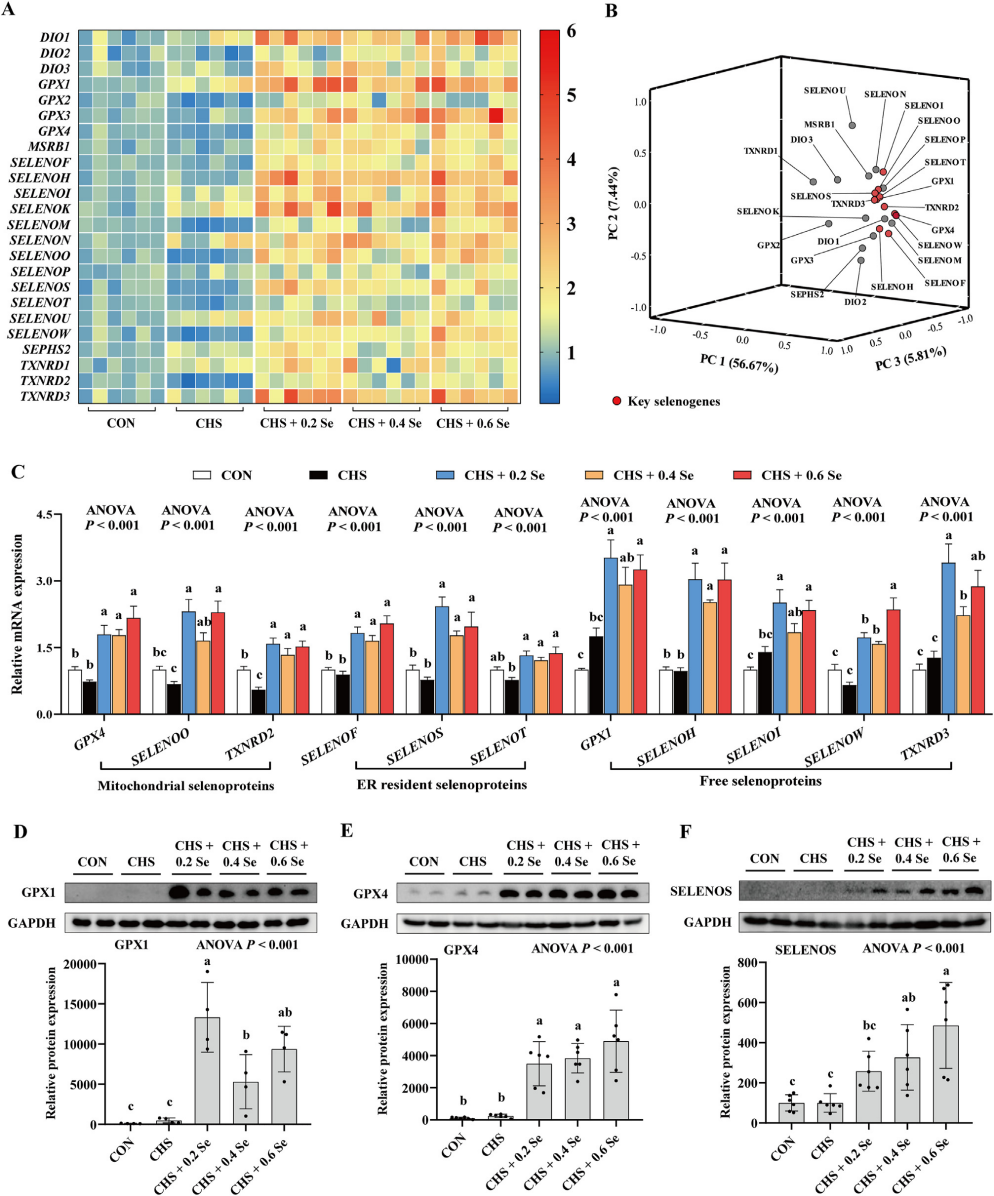
Expression of selenotranscriptome and key selenoproteins in breast muscle. (A) Heatmap of mRNA abundance of the selenotranscriptome. (B) Principal component analysis of the selenotranscriptome. (C) The relative mRNA expression of 11 key selenoproteins. (D) Relative protein expression of GPX1. (E) Relative protein expression of GPX4. (F) Relative protein expression of SELENOS. *DIO* = deiodinase; *GPX* = glutathione peroxidase; *MSRB1* = methionine sulfoxide reductase B1; *SELENO* = selenoprotein; *SEPHS2* = selenophosphate synthetase 2; *TXNRD* = thioredoxin reductase; ER = endoplasmic reticulum. CON = control group; CHS = chronic heat stress group; CHS + 0.2 Se = chronic heat stress + 0.2 Se mg/kg selenomethionine (SeMet) group; CHS + 0.4 Se = chronic heat stress + 0.4 Se mg/kg SeMet group; CHS + 0.6 Se = chronic heat stress + 0.6 Se mg/kg SeMet group. Results were expressed as mean ± SD (for the mRNA abundance of the selenotranscriptome, principal component analysis, mRNA expression of 11 key selenoproteins, protein expression of GPX4, protein expression of SELENOS, *n* = 6; for the protein expression of GPX1, *n* = 4). ^a-c^Different letters indicate significant differences (*P* < 0.05).

Selenium exerts its biological functions mainly through selenoproteins; here we determined the expression of the selenotranscriptome in breast muscle. Compared with the CON group, CHS group exhibited limited impact on the expression of the selenotranscriptome in breast muscle, except through down-regulating (*P <* 0.05) the expression of *TXNRD2* (Fig. 6A, detailed profiles are shown in Fig. S2). Dietary Se supplementation globally up-regulated the mRNA expression of the selenotranscriptome, except deiodinase 3 (*DIO3*), *SELENON*, selenoprotein P (*SELENOP*), selenoprotein U (*SELENOU*) and thioredoxin reductase 1 (*TXNRD1*), compared with CHS group.

We further performed a principal component analysis through the mathematical method of dimensionality reduction to distinguish the key selenogenes affected by CHS and SeMet. Eleven selenogenes were observed at relatively distant positions in three-dimensional space (Fig. 6B). Thus, these 11 genes (*GPX4*, *SELENOO*, *TXNRD2*, *SELENOF*, *SELENOS*, *SELENOT*, *GPX1*, selenoprotein H [*SELENOH*], selenoprotein I [*SELENOI*], *SELENOW*, thioredoxin reductase 3 [*TXNRD3*]) were the key selenogenes affected by CHS and SeMet. Among these key genes, 3 selenoproteins encoded by *GPX4*, *SELENOO*, *TXNRD2* are considered mitochondrial selenoproteins and 3 selenoproteins encoded by *SELENOF*, *SELENOS*, *SELENOT* are located in the ER. CHS exhibited limited impact on these key selenogenes, except for down-regulating (*P <* 0.05) the expression of *TXNRD2* (Fig. 6C). Dietary Se supplementation up-regulated these 11 key selenogenes, however, these changes did not show dose-dependence.

We validated 3 key selenoproteins (GPX1, GPX4 and SELENOS) at the protein level. CHS exhibited limited impact on the protein abundance of these 3 selenoproteins (Fig. 6D–F). Dietary Se increased (*P <* 0.05) the protein abundance of GPX1 and GPX4 (Fig. 6D and E) in a dose-independent manner, and linearly increased (*P* < 0.05) SELENOS in breast muscle of broilers exposed to CHS (Fig. 6F).

## 4 Discussion

Extreme heat events occur frequently around the world and HS has been a major factor restricting the broiler industry. HS causes poor growth performance and poor health status of broilers (Schirmann et al., 2021). For broilers, after 21 d, ADFI and ADG decrease 1.4% and 2.1% for each unit (°C) above the upper critical temperature (Schirmann et al., 2021). In this study, CHS increased the mortality and decreased the final body weight, ADFI and ADG, and increased FCR of broilers. CHS also reduced the breast muscle weight and breast muscle index. As skeletal muscle accounts for more than 50% of a broiler's body weight and breast muscle is the key component of a broiler's skeletal muscle, it is reasonable to presume that CHS causes skeletal muscle growth retardation in broilers. Selenium is considered to be an excellent antioxidant, and Se concentration in muscle is closely corrected with dietary Se levels in form of organic Se (Liu et al., 2021; Tang et al., 2021). Se deficiency induces nutritional muscular dystrophy and suppresses skeletal muscle growth in broilers (Yang et al., 2023). Our results showed that CHS exhibited limited impact on the Se concentration in breast muscle, while dietary SeMet supplementation linearly increased the Se levels in breast muscle. Likewise, additional Se supplementation, especially 0.4 and 0.6 mg Se/Kg, improved the growth performance, which increased the body weight and lowered the FCR of broilers. In addition, SeMet supplementation improved the breast muscle index. As an important biomarker, HSP70 is considered as a cellular thermometer and generally used to evaluate HS damage (Hartl et al., 2011). In present study, CHS increased the protein abundance of HSP70 in breast muscle of broilers, and the addition of Se decreased the protein expression of HSP70. Thus, organic Se supplementation in form of SeMet alleviates skeletal muscle injury and improves growth performance of broilers exposed to CHS.

Heat stress impairs meat quality (Gonzalez-Rivas et al., 2020), which generally leads to poor meat quality of broilers (Liu et al., 2022; Wen et al., 2019). In this study, CHS seriously compromised breast muscle meat quality of broilers, which increased the peak shear force, cooking loss, drip loss and 24 h *L∗* value, while lowering the pH and 24 h *a∗* value. The meat quality of skeletal muscle is closely related to the antioxidant capacity; under normal physiological conditions, excessive ROS are effectively eliminated by antioxidant substances such as GSH-Px and SOD (Zelko et al., 2002; Lubos et al., 2011), thus ensuring better meat quality. The reason CHS impairs meat quality is mainly due to the poor antioxidant capacity, which impairs redox status of skeletal muscle (Lu et al., 2017). Similarly, in this study, CHS disrupted redox homeostasis in breast muscle of broilers, which increased the ROS level and MDA level, and decreased the activity of GSH-Px. Current evidence reveals that there is a significant correlation between skeletal muscle Se concentration and meat quality (Li et al., 2011; Mahan et al., 1999). In the present study, dietary organic Se supplementation in the form of SeMet linearly increased the Se deposition in breast muscle, and improved breast muscle meat quality of broilers. As an excellent antioxidant, Se is the essential component of the antioxidant enzyme GSH-Px and it is actively involved in the antioxidant defense systems along with SOD (Rayman, 2012). Based on these results, dietary SeMet supplementation contributes to improvement in meat quality of broilers exposed to CHS through improving the antioxidant capacity of breast muscle.

HS-caused protein metabolism disorder may be the key factor that induces skeletal muscle growth retardation in broilers. Previous studies show that HS activates the caspase pathway, thus promoting protein degradation and inhibiting proliferation in swine skeletal muscle satellite cells (Gao et al., 2015). Besides, HS suppresses muscle hypertrophy and muscle protein synthesis in broilers though the mTOR pathway (Ma et al., 2018). Similarly, in the present study, CHS activated the caspase and ubiquitin proteasome system, and suppressed the mTOR pathway, which increased the mRNA expression of caspase 3, caspase 8, caspase 9, *UBA2*, *UBA3*, *UBE2A*, *UBE2B* and *UBE3A*, increased the activity of UBE1, increased the protein abundance of cleaved-caspase 3, and tended to decrease the protein abundance of P-4EBP1. The caspase family includes initiators such as caspase 8 and caspase 9 and executors such as caspase 3, caspase 6 and caspase 7; the initiator activates the executor, thus hydrolyzing intracellular protein (Julien and Wells, 2017). Activation of the caspase system promotes the degradation of myofibrillar proteins (Kemp and Wheeler, 2011). The ubiquitin-proteasome pathway also plays an important role in skeletal muscle protein degradation (Sandri, 2013). Ubiquitin molecules can activate UBE1 and transport it to ubiquitin-conjugating enzyme E2 (UBE2), then UBE2 and ubiquitin protein ligase E3 (UBE3) cooperate to bind ubiquitin molecules to proteins, thus degrading proteins (Gaczynska and Osmulski, 2018). Conversely, the mTOR pathway has gained attention as a key regulator of protein biosynthesis (Böhm et al., 2021). mTOR performs its functions mainly though regulating two downstream signaling molecules. On the one hand, mTOR phosphorylation inhibits 4EBP1, whereas on the other hand, phosphorylated mTOR activates S6K to promote the translation of ribosomal S6, thus promoting protein biosynthesis (Böhm et al., 2021; Xie et al., 2018). Therefore, our present results suggest that CHS promotes protein degradation and suppresses protein biosynthesis in the skeletal muscle of broilers. Dietary SeMet supplementation suppressed the caspase pathway and ubiquitin proteasome system, and promoted the mTOR pathway, especially through increasing the protein levels of P-4EBP1. Based on these results, SeMet improves protein metabolism homeostasis in the skeletal muscle of broilers under CHS though regulating the expression of 4EBP1, caspase and the ubiquitin proteasome system.

Activation of caspase and the ubiquitin proteasome system are generally mediated by mitochondrial dysfunction and ER stress (Li et al., 2016, 2019; Pradelli et al., 2010). Heat stress impairs mitochondrial function and causes ER stress in broilers (Zhang et al., 2018; Ma et al., 2022b). Mitochondrial dysfunction inhibits oxidative phosphorylation, increases the production of ROS and suppresses the synthesis of ATP (Chen et al., 2019). Meanwhile, excessive ROS activates caspase and the ubiquitin proteasome system (Chung et al., 2003; Nicolas and Frauke, 2018), thus promoting the degradation of proteins. In this study, CHS increased the ROS level and decreased the ATP level in breast muscle of broilers, which indicates that CHS causes mitochondrial dysfunction. We further explored the expression of mitochondrial stress biomarkers in breast muscle. HSP60 and CLPX are the representative biomarkers of mitochondrial stress. HSP60 mainly remained in the mitochondrial matrix, which removes excessive mitochondrial unfolded proteins under stress, thus maintaining mitochondrial homeostasis (Venkatesh and Suzuki, 2017). CLPX is associated with the respiratory chain complex II subunit succinate dehydrogenase B (SDHB) in mitochondria and loss of CLPX induces the accumulation of misfolded SDHB, thus impairing oxidative phosphorylation and activating autophagy (Seo et al., 2016). Mitochondrial dysfunction generally causes high levels of HSP60 and CLPX. In the present study, CHS increased the protein abundance of HSP60 and CLPX in breast muscle, which indicates that CHS causes mitochondrial dysfunction. Dietary SeMet supplementation recovered the levels of ROS, decreased the protein expression of HSP60 and CLPX, while increasing the levels of ATP. These results ulteriorly reveal that SeMet contributes to improvement in mitochondrial homeostasis in skeletal muscle of broilers exposed to CHS.

Under HS, excessive ROS can easily cause ER stress and ER dysfunction (Verfaillie et al., 2012). ER is the key organelle for protein processing and transportation. Generally, ER stress activates apoptosis and the ubiquitin proteasome system, thus inhibiting protein biosynthesis (Chen et al., 2019). ER-UPR is the representative behavior of the ER under stress (Jiwon and Ling, 2018). Under ER stress, excessive unfolded proteins accumulated in the ER facilitate the activation of ER-UPR through three ER transmembrane sensors: PERK, IRE1 and ATF6 (Calfon et al., 2002; Carrara et al., 2015; Ye et al., 2000). These sensors generally regulate multiple downstream signaling molecules such as eIF2α, activating transcription factor 4 (ATF4), C/EBP homologous protein (CHOP) and XBP-1, thus maintaining ER homeostasis. As an effector, GRP78 removes excessive unfolded proteins (Shen et al., 2002). Activation of ER-UPR promotes the accumulation of hypophosphorylated (active) eIF4E binding proteins, thus suppressing mRNA translation (Hamanaka et al., 2006). In present study, we found that CHS caused ER stress in skeletal muscle of broilers, which increased the mRNA expression of *PERK*, *eIF2α*, *IRE1*, *ATF6* and *GRP78*, and increased the protein expression of XBP-1 and GRP78. Broilers that received SeMet showed lower mRNA levels of *PERK* and *GRP78*, lower protein levels of XBP-1 and GRP78, and higher protein levels of P-4EBP1in breast muscle. Therefore, dietary SeMet contributes to the recovery of ER homeostasis in skeletal muscle of broilers under CHS, thus facilitating mRNA translation.

Accumulating evidence in different animal models indicates that Se performs its biological function mainly through selenoproteins (Jing et al., 2022; Tang et al., 2019, 2021). Generally, the expression of selenoproteins in animal muscle is effectively regulated by dietary Se levels (Sunde and Raines, 2011). We explored the mRNA expression of the selenotranscriptome in breast muscle of broilers under CHS. It was fascinating for us to find that although CHS alone exhibited limited impact on the expression of the selenotranscriptome, dietary SeMet supplementation globally up-regulated the expression of the selenotranscriptome. Eleven genes from the selenotranscriptome were identified as the key selenogenes affected by CHS and SeMet. Among them, GPX4, SELENOO and TXNRD2 are considered mitochondrial selenoproteins, which play a unique role in redox and mitochondrial homeostasis maintenance. GPX4 can effectively eliminate intracellular lipid peroxides (Schnurr et al., 1996). SELENOO and TXNRD2 protect cells against disulfide damage and regulate redox homeostasis (Han et al., 2014; Santesmasses et al., 2020). SELENOF, SELENOS and SELENOT are located in the ER and contribute to the homeostasis of the ER (Pitts and Hoffmann, 2018). SELENOS and SELENOF participate in protein quality control by mediating disulfide bond formation, thus contributing to the scavenging of unfolded or misfolded proteins under ER stress (Emer et al., 2009; Ren et al., 2018). SELENOT benefits ER homeostasis though scavenging excessive ROS and suppressing apoptosis (Huang et al., 2020). Though the other five selenoproteins (GPX1, SELENOH, SELENOI, SELENOW and TXNRD3) are in a free state, they also play important roles in cellular stability maintenance. GPX1 clears excessive ROS and maintains cell metabolic homeostasis (Sun et al., 2020). SELENOH participates in redox regulation, while it is also a key regulator for cell cycle progression (Bertz et al., 2018). Though SELENOI does not catalyze redox reactions, SELENOI deficiency results in lower cell proliferation capacity (Ma et al., 2021). Similar to TXNRD2, TXNRD3 is a small protein that plays a key role in the cellular redox regulatory system, and SELENOW synergistically works with them (Kang et al., 2021). Besides, SELENOW participates in the proliferation and differentiation of myoblasts (Li et al., 2011). The enhancement of water-holding capacity of meat is associated with the increasing expression of SELENOW (Loflin et al., 2006). Therefore, up-regulation of those selenogenes may contribute to redox and mitochondrial homeostasis under CHS. Additionally, we assayed 3 major selenoproteins and found that SeMet supplementation also increased the protein levels of GPX1, GPX4 and SELENOS. Based on the above results, dietary SeMet supplementation can linearly increase the Se deposition in muscle, which allows muscle to quickly mobilize Se to synthesize selenoproteins under stress. Up-regulation of these selenoproteins, especially the 11 key selenoproteins, contributes to improving antioxidant capacity and relieving mitochondrial dysfunction and ER stress, thus improving meat quality and skeletal muscle growth in broilers exposed to CHS.

## 5 Conclusion

Our results in the present study reveal that CHS-induced skeletal muscle damage in broilers is accompanied by mitochondrial dysfunction and ER stress, which lower antioxidant capacity and suppress protein biosynthesis, thus causing skeletal muscle growth retardation and poor meat quality. Dietary organic Se supplementation in the form of SeMet linearly increases the Se deposition in skeletal muscle, which contributes to increases in the expression of skeletal muscle selenoproteins and mitigates the negative effects of CHS. The increased expression of selenoproteins alleviates mitochondrial dysfunction and ER stress, improves the antioxidant capacity, and improves protein biosynthesis and suppresses caspase and ubiquitin proteasome system, thus improving the growth performance and breast muscle meat quality of broilers. Several key selenoproteins such as GPX4, SELENOO, TXNRD2, SELENOF, SELENOS, SELENOT, GPX1, SELENOH, SELENOI, SELENOW and TXNRD3 play important roles in this process.

## Author contributions

**Jinzhong Jing**: Investigation, Methodology, Experiment design, Data analysis, Writing - original draft. **Jiayi Wang**: Investigation, Methodology, Data analysis, Validation. **Xiaoyu Xiang**: Sample collection, Supervision. **Shenggang Yin**: Validation, Sample collection, Supervision. **Jiayong Tang**: Validation, Supervision, Review and editing. **Longqiong Wang**: Validation, Sample collection, Supervision. **Gang Jia**, **Guangmang Liu**, **Xiaoling Chen**, **Gang Tian**, **Jingyi Cai**, **Bo Kang** and **Lianqiang Che**: Validation, Supervision. **Hua Zhao**: Investigation, Methodology, Review and editing, Funding acquisition.

## Declaration of competing interest

We declare that we have no financial and personal relationships with other people or organizations that can inappropriately influence our work, and there is no professional or other personal interest of any nature or kind in any product, service and/or company that could be construed as influencing the content of this paper.
