# Supplementary material for: Selenomethionine alleviates chronic heat stress-induced breast muscle injury and poor meat quality in broilers via relieving mitochondrial dysfunction and endoplasmic reticulum stress
Source: Anim Nutr. 2024 Feb 1;16:363–75. doi: 10.1016/j.aninu.2023.12.008 (PMC10867585; doi:10.1016/j.aninu.2023.12.008)
Supplement: Multimedia component 1 [file mmc1.docx]

**Table S1 Composition and nutrient levels of the basal diet (% dry matter).**

| Item | 0–3 Week | 4–6 Week |
| --- | --- | --- |
| Ingredients |  |  |
| Corn (CP 8%) | 58.39 | 62.50 |
| Soybean meal (CP 44.2%) | 33.05 | 31.30 |
| Fish meal (CP 62.5%) | 2.00 | 0.00 |
| Soybean oil | 2.50 | 2.50 |
| CaCO_3_ | 1.30 | 1.20 |
| CaHPO_4_ | 1.65 | 1.70 |
| NaCl | 0.40 | 0.30 |
| Lys-HCl | 0.08 | 0.02 |
| DL-Met | 0.26 | 0.17 |
| L-Thr | 0.02 | 0.01 |
| Choline chloride | 0.15 | 0.10 |
| Premix ^1^ | 0.20 | 0.00 |
| Premix ^2^ | 0.00 | 0.20 |
| Total | 100.00 | 100.00 |
| Nutrient composition ^3^ | | |
| Metabolic energy, MJ/kg | 12.29 | 12.43 |
| Crude protein | 20.87 | 19.03 |
| Total Ca | 1.01 | 0.91 |
| Total P | 0.72 | 0.67 |
| Available P | 0.45 | 0.40 |
| Lys | 1.19 | 1.00 |
| Met | 0.58 | 0.46 |
| Met + Cys | 0.91 | 0.77 |
| Thr | 0.81 | 0.72 |
| Val | 0.95 | 0.87 |
| Trp | 0.23 | 0.21 |

^1^ Premix for 0–3 wk broiler provided (per kilogram): Cu (CuSO_4_·5H_2_O), 8 mg; Fe (FeSO_4_·7H_2_O), 100 mg; Mn (MnSO_4_·H_2_O), 120 mg; Zn (ZnSO_4_·H_2_O), 100 mg; I (KI), 0.7 mg; vitamin A, 8000 IU; vitamin D_3_, 2000 IU; vitamin E, 20 IU; vitamin K_3_, 3.2 mg; vitamin B_1_, 2 mg; vitamin B_2_, 6.4 mg; vitamin B_6_, 4 mg; vitamin B_12_, 0.2 mg; D-biotin, 1.1 mg; D-pantothenic acid, 12 mg; folic acid, 1 mg; nicotinamide, 40 mg.

^2^ Premix for 4–6 wk broiler provided (per kilogram): Cu (CuSO_4_·5H_2_O), 8 mg; Fe (FeSO_4_·7H_2_O), 80 mg; Mn (MnSO_4_·H_2_O), 100 mg; Zn (ZnSO_4_·H_2_O), 80 mg; I (KI), 0.7 mg; vitamin A, 6000 IU; vitamin D_3_, 1500 IU; vitamin E, 15 IU; vitamin K_3_, 2.4 mg; vitamin B_1_, 1.5 mg; vitamin B_2_, 4.8 mg; vitamin B_6_, 3 mg; vitamin B_12_, 0.15 mg; D-biotin, 0.825 mg; D-pantothenic acid, 9 mg; folic acid, 0.75 mg; nicotinamide, 30 mg.

^3^ The nutrient levels (crude protein, total Ca, total P) are measured values. The metabolic energy are calculated values.

**Table S2 Primers used for the RT-PCR.**

| Gene | NCBI Sequence | Sequence | | Product size, bp |
| --- | --- | --- | --- | --- |
| β-Actin | NM_205518.2 | F: | TATTGCTGCGCTCGTTGTTG | 52 |
|  |  | R: | GAAACCGGCCTTGCACATAC |  |
| Caspase 3 | NM_204725.2 | F: | AGGTGGAGGAGCTCTCCTATG | 199 |
|  |  | R: | CCTGAGCGTGGTCCATCTTT |  |
| Caspase 8 | NM_204592.4 | F: | CCGATTCTCTGGGCAACTGT | 66 |
|  |  | R: | GGGGTCGGCTGGTCATTTTA |  |
| Caspase 9 | XM_046931415.1 | F: | ACTGGAACATTACGCCCGTT | 94 |
|  |  | R: | CGGGATCTGCTTGTACCTCC |  |
| *UBA2* | NM_001030571.3 | F: | CCCGTCGCCGTTTTTCG | 174 |
|  |  | R: | CAATCACGTCGATGTTGCTGA |  |
| *UBA3* | XM_040682464.2 | F: | GCGGATGGTGAGGAACCAAT | 52 |
|  |  | R: | CTCCAGAGTCCCCATACCCA |  |
| *UBE2A* | NM_204865.2 | F: | GACCGCTCCCGTTCGTT | 94 |
|  |  | R: | CGAATCACTCATACACCGCC |  |
| *UBE2B* | XM_040682998.2 | F: | CCTCCTTGGGGAGCGTAG | 74 |
|  |  | R: | TTACAAACACCCCTCTCCCTC |  |
| *UBE3A* | XM_040658102.2 | F: | CTAGCCGAATAAAGGAACGAGC | 57 |
|  |  | R: | TGGGCACCTTTGGAGTTGTT |  |
| *mTOR* | XM_040689168.2 | F: | ATGGCTTCCAAGGCTATCGG | 89 |
|  |  | R: | TCCAGAGCACGTTTCACCTC |  |
| *4EBP1* | XM_040689367.2 | F: | CGGGCGGAACCAGGATTATT | 59 |
|  |  | R: | CGGAGAATTACGGCACTCCA |  |
| *S6K1* | NM_001030721.2 | F: | TTCACACCCGCGCTATGG | 80 |
|  |  | R: | CTCCAGCTCCTCATCCGAAC |  |
| *PERK* | XM_040671515.2 | F: | GTGGCTGACTGGAAGGTTATGG | 59 |
|  |  | R: | CAATTGGAGTGCAAAACTGGTATTC |  |
| *ATF4* | NM_204880.3 | F: | CCACACGTTACCGGCAAAA | 59 |
|  |  | R: | AAATCTCTGCACTCCCCTGACA |  |
| *eIF2α* | NM_001031323.2 | F: | AAACCTGCGCAAGCGTATAGAC | 59 |
|  |  | R: | CCTCATGAAGCTTGGAACTTGTTAC |  |
| *CHOP* | KP064314.1 | F: | TGCCGTGCTTAGCAGAATGG | 94 |
|  |  | R: | ATGCTGTACAGTGGTGCTGGAA |  |
| *IRE1* | NM_001285499.2 | F: | GGCTGTGTCTTTTATTATGTGGTATCTG | 59 |
|  |  | R: | TGGCTTGTCGCTGTAGAGATTTG |  |
| *XBP-1* | NM_001006192.2 | F: | CCATTCCTGACAACCTCCACAT | 60 |
|  |  | R: | CGAACAGGAGATCAGACTCAGAATC |  |
| *ATF6* | XM_040677276.2 | F: | ATGCAGAGCTCCGTTAGTCAGAA | 59 |
|  |  | R: | CTGAGGCTCGTGTCTGTGTAAGG |  |
| *GRP78* | NM_205491.2 | F: | CGGACGATGAGGAGAAAAAGG | 60 |
|  |  | R: | GAATAGGTGGTACCGAGGTCGAT |  |
| *DIO1* | NM_001097614.2 | F: | TCTACAAGGGAGGAGTGGGG | 62 |
|  |  | R: | TTCCAGGACAGCGCGTATTT |  |
| *DIO2* | NM_001324555.3 | F: | TGCGCGCGGTCAAACTT | 64 |
|  |  | R: | TTGCCCTTGGCTATGTGGATT |  |
| *DIO3* | NM_001122648.3 | F: | CAGTACAAAACCCGGCTCCA | 50 |
|  |  | R: | TACACTTGGATGACCACCGC |  |
| *GPX1* | NM_001277853.3 | F: | ACCATGTTCGAGAAGTGCGA | 65 |
|  |  | R: | TCTCTCAGGAAGGCGAACAG |  |
| *GPX2* | NM_001277854.3 | F: | GGTCCTCATCGAGAACGTGG | 59 |
|  |  | R: | GCTGGGTGTAATCCCTCACC |  |
| *GPX3* | NM_001163232.3 | F: | CACCATCTACGACTACGGGG | 74 |
|  |  | R: | TCTTCCCCGCGTACTTTCTG |  |
| *GPX4* | NM_001346449.2 | F: | GTGATGCTCCCCTTCGTCTC | 56 |
|  |  | R: | TACAGGTAGGCGGGCAGAT |  |
| *MSRB1* | NM_001135558.3 | F: | CCCGCAGGAGAATTAAGCGA | 76 |
|  |  | R: | GCTCCCATACACCCTGACTG |  |
| *SELENOF* | NM_001012926.3 | F: | AGTACGTGCGTGGTTCTGAC | 58 |
|  |  | R: | GCAATGTTCCCACTGTCGTC |  |
| *SELENOH* | NM_001277865.2 | F: | GCCGTAGAGATCAACCCGC | 50 |
|  |  | R: | GACACCTCGAAGCTGTTCCT |  |
| *SELENOI* | NM_001031528.4 | F: | TGCCAGCCTCTGAACTGGAT | 69 |
|  |  | R: | TGCAAACCCAGACATCACCAT |  |
| *SELENOK* | NM_001025441.2 | F: | ATAAATCACTGGGGTGGAGGC | 93 |
|  |  | R: | GCTCCTTTGCCTGCTTCTTAC |  |
| *SELENOM* | NM_001277859.2 | F: | ACATCCCGCTGTACCATAACCT | 126 |
|  |  | R: | TCTCCTCCCGGGTCATGTC |  |
| *SELENON* | NM_001114972.3 | F: | CTGTATGGGGCGAGTGAAGG | 99 |
|  |  | R: | TATCACAGAGGGGACCGAGG |  |
| *SELENOO* | NM_001115017.5 | F: | CCCAGCGTTAACCGGAATGA | 147 |
|  |  | R: | TCTTGCCGTCCGCTTTGTTA |  |
| *SELENOP* | NM_001031609.3 | F: | CCAAGTGGTCAGCATTCACATC | 81 |
|  |  | R: | ATGACGACCACCCTCACGAT |  |
| *SELENOS* | NM_001024734.3 | F: | CCGACATGGTGGTAAGAAGACA | 76 |
|  |  | R: | GCTTGTGCATTCAACTCCTCTTG |  |
| *SELENOT* | NM_001006557.4 | F: | GATCTGCGTCTCCTGAGGTT | 97 |
|  |  | R: | GTAGTTCTCCCCCTCGATGC |  |
| *SELENOU* | NM_001193519.3 | F: | GGCTGCTTCGGAAATGTCT | 95 |
|  |  | R: | CTGTTATGGCTGCGCCAAC |  |
| *SELENOW* | NM_001166327.2 | F: | TGTGGGTCTGCTTTACGCC | 70 |
|  |  | R: | AAGCTGGAAGGTGCAAAATGAA |  |
| *SEPHS2* | NM_001366334.2 | F: | AGGTTGACGAGTTACAGCGA | 51 |
|  |  | R: | CTCCGGCACTTTACAGCCTC |  |
| *TXNRD1* | NM_001030762.4 | F: | GCCTTACTGTCCGGGGAAAA | 63 |
|  |  | R: | CCTGCACATTCCAAGGCAAC |  |
| *TXNRD2* | NM_001122691.3 | F: | AAAGATGCCCAGCACTACGG | 71 |
|  |  | R: | GCTTGAGCCATCACAGACCA |  |
| *TXNRD3* | NM_001122777.3 | F: | TGACCTCTTCTCCCTGCCTTA | 53 |
|  |  | R: | AAGCACCCACAACTAGCGTT |  |

*UBA2* = ubiquitin-like modifier activating enzyme 2; *UBA3* = ubiquitin-like modifier activating enzyme 3; *UBE2A* = ubiquitin conjugating enzyme E2 A; *UBE2B* = ubiquitin conjugating enzyme E2 B; *UBE3A* = ubiquitin protein ligase E3 A; *mTOR* = mammalian target of rapamycin; *4EBP1* = eukaryotic translation initiation factor 4E (eIF4E)-binding protein 1; *S6K1* = mitogen-stimulated protein kinase p70 ribosomal protein S6 kinase 1; *PERK* = protein kinase-like endoplasmic reticulum kinase; *ATF4* = activating transcription factor 4; *eIF2α* = eukaryotic initiation factor-2α; *CHOP* = C/EBP homologous protein; *IRE1* = inositol-requiring protein 1; *XBP-1* = X-box binding protein 1; *ATF6* = activating transcription factor 6; *GRP78* = glucose regulatory protein 78; *DIO* = deiodinase; *GPX* = glutathione peroxidase; *SELENO* = selenoprotein; *TXNRD* = thioredoxin reductase;

**Table S3 Primary antibodies for the** **western blot analyses.**

| Antibody | Company | Address | Commodity code | Dilution ratio |
| --- | --- | --- | --- | --- |
| P-4EBP1 | Cell signaling technology | Massachusetts, USA | 2855 | 1:1000 |
| 4EBP1 | Cell signaling technology | Massachusetts, USA | 9644 | 1:1000 |
| P-S6K1 | Cell signaling technology | Massachusetts, USA | 9209 | 1:1000 |
| S6K1 | Cell signaling technology | Massachusetts, USA | 9202 | 1:1000 |
| GPX1 | Zen BioScience | Chengdu, China | R26805 | 1:500 |
| CLPX | Zen BioScience | Chengdu, China | R23947 | 1:1000 |
| XBP-1 | Zen BioScience | Chengdu, China | R27438 | 1:1000 |
| P-eIF2α | Zen BioScience | Chengdu, China | 310073 | 1:500 |
| eIF2α | Zen BioScience | Chengdu, China | 340347 | 1:500 |
| GPX4 | Zen BioScience | Chengdu, China | 513309 | 1:2000 |
| Caspase3 | Proteintech Group | Illinois, USA | 19677-1-AP | 1:500 |
| GRP78 | Proteintech Group | Illinois, USA | 66574-1-Ig | 1:5000 |
| HSP60 | Proteintech Group | Illinois, USA | 66041-1-Ig | 1:5000 |
| HSP70 | Proteintech Group | Illinois, USA | 10995-1-AP | 1:5000 |
| SELENOS | Proteintech Group | Illinois, USA | 15591-1-AP | 1:1000 |
| GAPDH | Proteintech Group | Illinois, USA | 60004-1-Ig | 1:10000 |

4EBP1 = eukaryotic translation initiation factor 4E (eIF4E)-binding protein 1; S6K1 = mitogen-stimulated protein kinase p70 ribosomal protein S6 kinase 1; GPX1 = glutathione peroxidase 1; CLPX = ATP-dependent CLP protease ATP-binding subunit; XBP-1 = X-box binding protein 1; eIF2α = eukaryotic initiation factor-2α; GPX4 = glutathione peroxidase 4; GRP78 = glucose regulatory protein 78; HSP60 = heat shock protein 60; HSP70 = heat shock protein 70; SELENOS = selenoprotein S; GAPDH = glyceraldehyde-3-phosphate dehydrogenase.


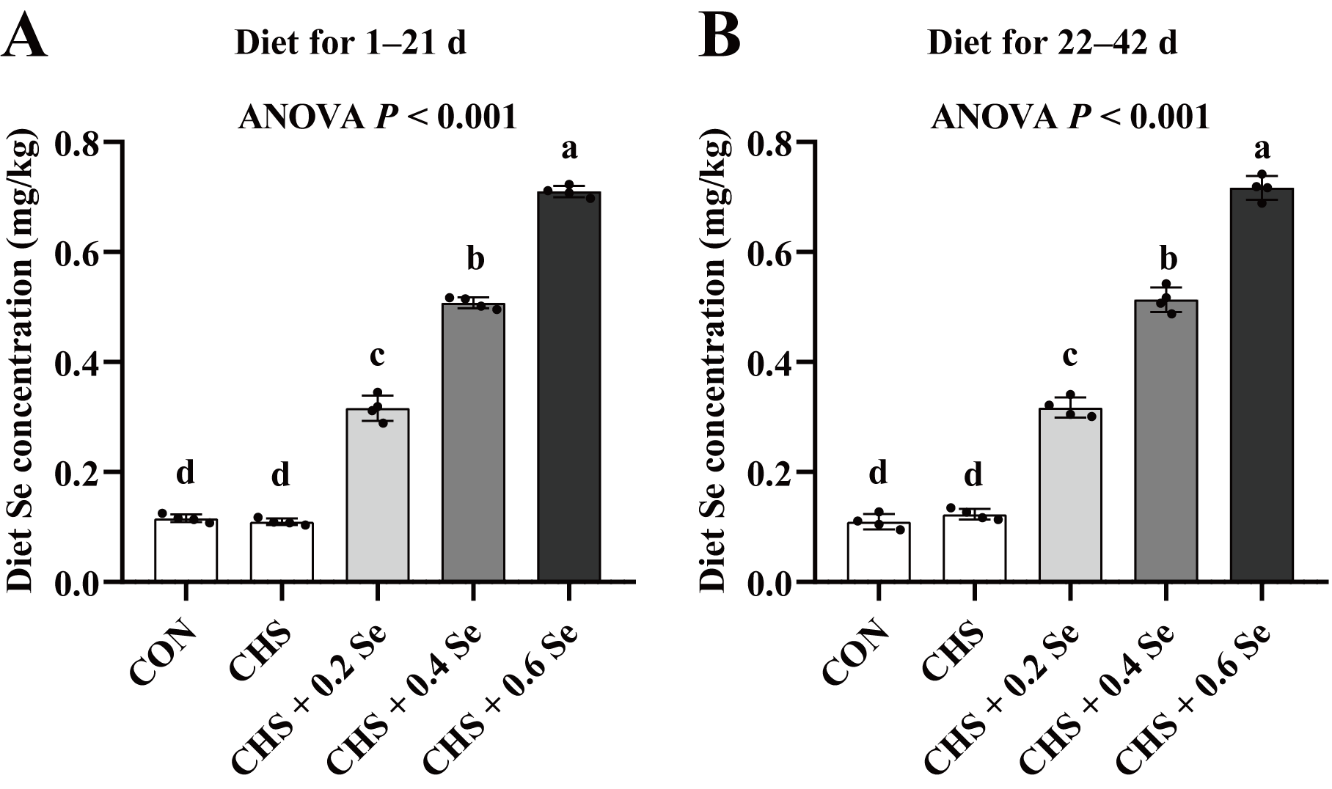


**Fig. S1 Se concentration in diet.** Results were expressed as mean ± SD (*n* = 4). CHS = chronic heat stress group; CHS + 0.2 Se = chronic heat stress + 0.2 Se mg/kg selenomethionine (SeMet) group; CHS + 0.4 Se = chronic heat stress + 0.4 Se mg/kg SeMet group; CHS + 0.6 Se = chronic heat stress + 0.6 Se mg/kg SeMet group. ^a-d^Different letters indicate significant differences (*P* < 0.05).


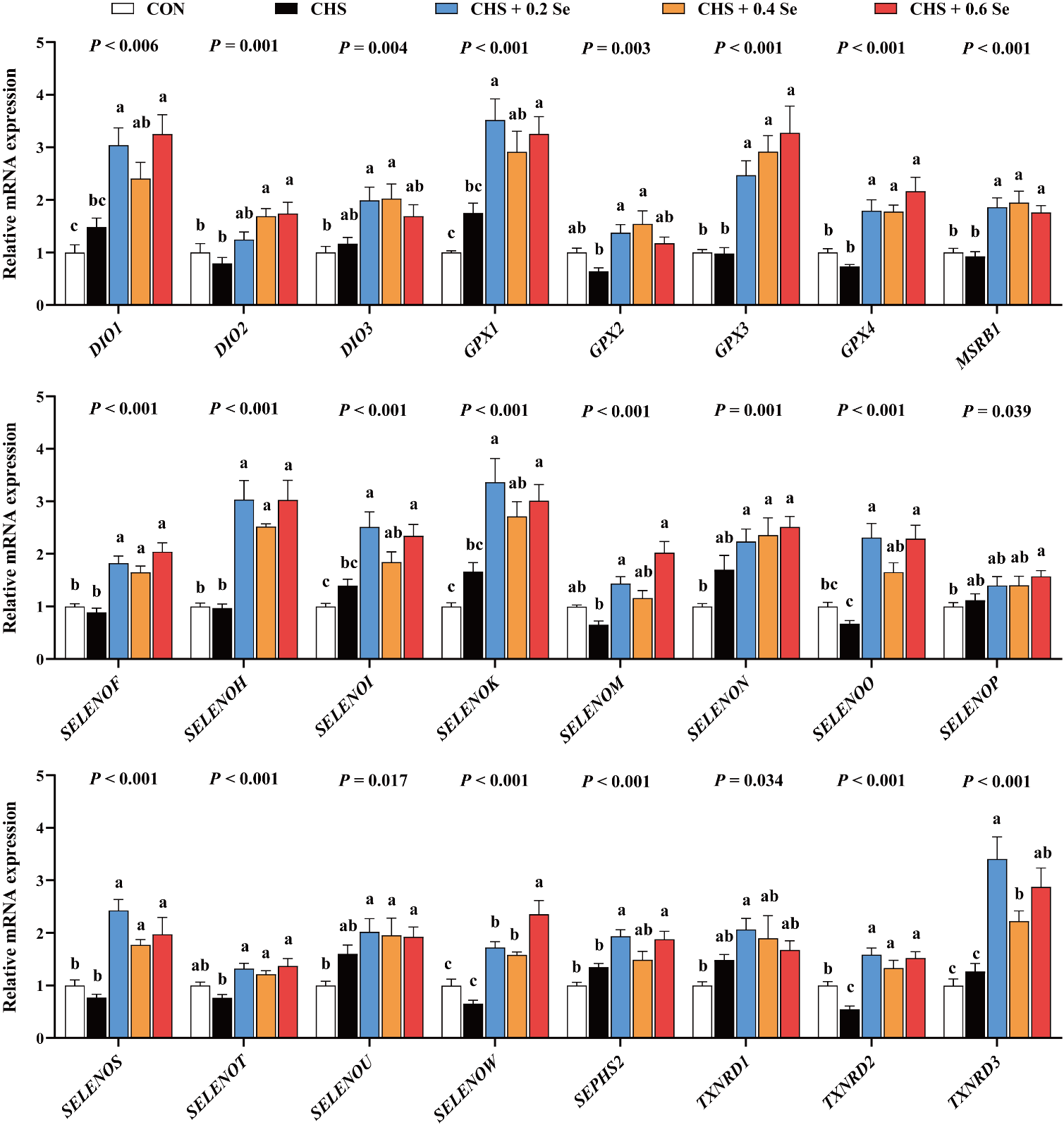


**Fig. S2 The expression of selenotranscriptome in breast muscle.** *DIO* = deiodinase; *GPX* = glutathione peroxidase; *MSRB1* = methionine sulfoxide reductase B1; *SELENO* = selenoprotein; *SEPHS2* = selenophosphate synthetase 2; *TXNRD* = thioredoxin reductase. Results were expressed as mean ± SD (*n* = 6). CHS = chronic heat stress group; CHS + 0.2 Se = chronic heat stress + 0.2 Se mg/kg selenomethionine (SeMet) group; CHS + 0.4 Se = chronic heat stress + 0.4 Se mg/kg SeMet group; CHS + 0.6 Se = chronic heat stress + 0.6 Se mg/kg SeMet group. ^a-c^Different letters indicate significant differences (*P* < 0.05).
